# CD155 as a therapeutic target in alveolar echinococcosis: insights from an *Echinococcus multilocularis* infection mouse model

**DOI:** 10.3389/fmicb.2025.1624387

**Published:** 2025-07-01

**Authors:** Xue Zhang, Liang Li, Tao Sun, Ning Yang, Hui Liu, Jin Chu, Junlong Xue, Guodong Lü, Tuerganaili Aji, Xiaojuan Bi, Renyong Lin

**Affiliations:** ^1^State Key Laboratory of Pathogenesis, Prevention and Treatment of High Incidence Diseases in Central Asia, Clinical Medical Research Institute, The First Affiliated Hospital of Xinjiang Medical University, Urumqi, China; ^2^Xinjiang Key Laboratory of Echinococcosis, Clinical Medical Research Institute, The First Affiliated Hospital of Xinjiang Medical University, Urumqi, China; ^3^Department of Hepatobiliary and Hydatid Diseases, Digestive and Vascular Surgery Center, The First Affiliated Hospital of Xinjiang Medical University, Urumqi, China

**Keywords:** *Echinococcus multilocularis*, CD155, T-cell exhaustion, alveolar echinococcosis, metacestode vesicles

## Abstract

**Introduction:**

Alveolar echinococcosis (AE) is a life-threatening zoonotic parasitic disease caused by the metacestode stage of *Echinococcus multilocularis*, characterized by granulomatous lesions and liver fibrosis. Immune exhaustion is the key mechanism by which *E. multilocularis* evades host immune responses and maintains long-term parasitism. Although CD155 is recognized as an immune checkpoint molecule, its specific role and underlying mechanism in AE remain unclear.

**Methods:**

A mouse model of *E. multilocularis* infection was used to investigate the role of CD155 in AE progression. Flow cytometry, immunohistochemistry, and immunofluorescence were employed to assess CD155 expression and analyze T-cell function. In addition, liver weight, lesion size, lesion number, inflammation index, collagen deposition (via Masson staining), and stellate cell activation (via α-SMA immunohistochemistry) were statistically quantified in the CD155 hepatocyte knockout mice. Each experimental group included five mice (*n* = 5).

**Results:**

CD155 expression in hepatocytes was significantly increased—approximately 2-fold compared to Sham controls—and predominantly localized near lesion sites. The infected group showed significantly reduced percentages of functional CD4^+^IFN-γ^+^, CD4^+^CD107a^+^, and CD8^+^CD107a^+^ T cells (*p* < 0.05), along with enrichment of exhausted TIGIT^+^ T cells adjacent to CD155^+^ hepatocytes. *In vitro*, CD155 expression in hepatocytes was upregulated in a dose-dependent manner when co-cultured with metacestode vesicles, reaching 1.5-fold that of the control. Notably, hepatocyte-specific CD155 knockout in infected mice restored CD4^+^ and CD8^+^ T-cell function and reduced liver damage, as indicated by decreased lesion burden.

**Conclusion:**

In the *E. multilocularis* infection mouse model, excretory/secretory products from metacestode vesicles upregulated CD155 expression in hepatocytes, contributing to an immunosuppressive microenvironment and T-cell exhaustion. Targeting CD155 reverses this immunosuppression and mitigates hepatic pathology, highlighting CD155 as a promising therapeutic target for AE.

## 1 Introduction

Alveolar Echinococcosis (AE) is a life-threatening parasitic disease caused by the metacestode (larval) form of *Echinococcus multilocularis* (*E. multilocularis*), prevalent in Xinjiang and other northwestern regions of China (Budke et al., 2017). The lesions primarily affect the liver and are predominantly characterized by multilocular vesicle masses consisting of numerous interconnected round or oval vesicles, with diameters ranging from 0.1 to 0.7 cm. These vesicles contain transparent fluid and a substantial number of germinal layer cells, which can proliferate predominantly through continuous exogenous budding in an outward direction. Notably, the outer horn cortex of the vesicles is thin and frequently incomplete, leading to the absence of a fibrous tissue capsule that separates the vesicles from the host tissue. Consequently, the continuous stimulation of metacestode vesicle products damages the host’s liver, leading to chronic inflammation and secondary granulomas formation around the lesions. This persistent liver damage may ultimately result in severe liver diseases, including liver failure and potential death (Baumann et al., 2019; Gottstein et al., 2017; Wei et al., 2018).

Immune exhaustion refers to the phenomenon of impaired T-cell effector function in cancer or chronic infection (Salnikov et al., 2023). Exhausted T cells are characterized by elevated and persistent expression of inhibitory molecules, such as CTLA-4, PD-1, TIGIT, and LAG3 (Galon and Bruni, 2019; Butler et al., 2011). Recent studies have demonstrated that infection with *E. multilocularis* is associated with T-cell exhaustion, characterized by increased expression of immune checkpoints, including TIGIT and PD-1 (Zhang et al., 2024). These receptors restrict the activity of lymphocytes, resulting in an impaired host immune response. As a result, this impairment allows the parasite to evade the host’s immune attack, thereby facilitating its long-term parasitism. Accordingly, reversing immune exhaustion is a crucial therapeutic strategy for eliminating the parasite and mitigating AE progression.

CD155 is a cell adhesion molecule classified within the immunoglobulin superfamily and characterized as a type I transmembrane glycoprotein. Initially identified as a poliovirus receptor, it is now increasingly recognized as a potential target for immunotherapy due to its role in immune regulation (Liu et al., 2023). Numerous studies have confirmed that CD155 overexpression in tumors serves not only as a direct indicator of tumor progression but is also closely correlated with poor prognosis in patients (Qu et al., 2015; Xu et al., 2020; Hai et al., 2020). CD155 is generally expressed at moderate to low levels in healthy human tissues; however, its expression is significantly upregulated in various tumors (Nishiwada et al., 2015; Iguchi-Manaka et al., 2016; Bevelacqua et al., 2012; Nakai et al., 2010; Zheng et al., 2020; Huang et al., 2017; Merrill et al., 2004; Atsumi et al., 2013; Zhao et al., 2019; Bates et al., 2018). This increased expression facilitates immune exhaustion through the CD155/TIGIT pathway, thereby accelerating tumor progression by inhibiting antitumor immune responses (Yue et al., 2023). Our previous study demonstrated a substantial increase in the hepatic expression of CD155 during the course of AE (Zhang et al., 2023). However, the specific mechanisms and roles linked to the increased expression of CD155 under these conditions remain unclear. This study demonstrated that excretory/secretory products from metacestode vesicles facilitate CD155 overexpression in hepatocytes, which contributes to the exhaustion of surrounding T cells in AE. Targeting CD155 has the potential to reverse T-cell exhaustion under these conditions, indicating that CD155 may serve as a significant therapeutic target for treating AE.

## 2 Materials and methods

### 2.1 Animals

C57BL/6 mice (aged 7–8 weeks), CD155^f/f^ mice, and Alb-CreERT2 mice were bought from Nanmo Biotechnology (Shanghai, China). To generate tamoxifen-inducible hepatocyte CD155 knockout mice, CD155^f/f^ mice were crossed with Alb-CreERT2 mice. At 8 weeks of age, these mice received intraperitoneal injections of tamoxifen (75 mg/kg/day, dissolved in corn oil, 160 μL per mouse) every other day for two weeks to induce Cre-mediated recombination, resulting in the conditional knockout of CD155 in hepatocytes (CD155_Alb_^−/−^ mice). The outcomes of mouse genotype identification and CD155 expression in the hepatocytes of CD155_Alb_^−/−^ mice are illustrated in Supplementary Figure S1. The construction of the *E. multilocularis* infection mouse model was performed in accordance with established protocols in the field (Zhang et al., 2024). There were five mice in each experimental group, including the Sham group, the *E. multilocularis*-infected (Em) group, CD155^f/f^ group, and CD155_Alb_^−/−^ group. All mice were maintained in an SPF environment, and all animal experiments were conducted in compliance with the management regulations of the Ethics Committee of the First Affiliated Hospital of Xinjiang Medical University (No. K202208-15).

### 2.2 Protoscoleces and cell culture

Protoscoleces were cultured in sterile culture bottles at 37°C with 5% CO_2,_ and the culture medium was replaced every 3 days. The culture medium consisted of RPMI-1640 medium, fetal bovine serum, 5% yeast extract, 30% glucose, 1% penicillin, and 1% streptomycin. At various time points during the culture (days 1, 7, 15, and 30), samples were randomly taken under a microscope to observe, photograph, and record until the protoscoleces developed into metacestode vesicles.

Metacestode vesicles were co-cultured with HL-7702 cells for 24 h in 6-well plates containing 24-mm diameter transwells equipped with a 0.4 μm porous polystyrene membrane. The metacestode vesicles were introduced into the upper compartment of the transwell system, while HL-7702 cells were cultured in the lower compartment. Following the 24-h co-culture, CD155 expression in HL-7702 cells was analyzed using flow cytometry. HL-7702 cells cultured alone under identical conditions served as the control group for this experiment to assess the basal expression level of CD155.

### 2.3 Hematoxylin and eosin and Masson’s trichrome staining

For histological examination, liver tissues were fixed in 4% paraformaldehyde buffer, followed by paraffin embedding and sectioning into 4-μm slices. The slides were deparaffinized with xylene and rehydrated through graded ethanol solutions. Subsequently, 3% H_2_O_2_ was used to eliminate endogenous peroxidase activity. Subsequently, separate sections were stained with hematoxylin and eosin (H&E) for general histological evaluation, or with Masson’s trichrome to assess collagen deposition, followed by dehydration through graded ethanol. Images were captured using an optical microscope. The inflammatory index was assessed by counting the number of inflammatory cells within the lesion area.

### 2.4 Immunohistochemistry

For immunohistochemistry analysis, after the sections were dewaxed and hydrated, 3% H_2_O_2_ was used to block peroxidase activity. Antigen retrieval was performed using citrate buffer under microwave thawing conditions. Nonspecific staining was blocked with 10% goat serum, and the samples were incubated overnight with a primary antibody at 4°C (anti-mouse α-SMA, 1:500; Abcam; anti-mouse CD155, 1:50; R&D Systems). The next day, the membranes were incubated with the secondary antibody. A DAB display kit was used for color development, followed by H&E staining and neutral gum sealing. Images were observed under a microscope at 200 × magnification and captured using the principle of random selection of visual fields, with 5 visual fields per slide. The collected images were analyzed using Image-Pro Plus 6.0 software.

### 2.5 Flow cytometry

Single-cell suspensions were prepared and adjusted to a concentration of 1 × 10^6^ cells in 100 μL. The cells were cultured with a stimulant in an incubator at 37°C for 4 h. Subsequently, an anti-CD16/CD32 antibody was added and incubated on ice for 20 min to block Fc receptors, followed by staining with external antibodies. The membrane was permeabilized, and an internal standard antibody was introduced. After centrifugation in PBS, data were acquired using a flow cytometer and analyzed with FlowJo software (version 7.6.1).

### 2.6 Statistical analysis

Statistical Package for the Social Sciences software (version 20.0) was used for statistical analysis. Normally distributed measurement data are expressed as means ± standard deviation. Comparison between two groups was performed using *t*-test, and comparison among multiple groups was performed using one-way ANOVA followed by Tukey post-test, with *p* < 0.05 considered statistically significant.

## 3 Results

### 3.1 *Echinococcus multilocularis* infection upregulates hepatocyte CD155 expression

**Figure 1:**
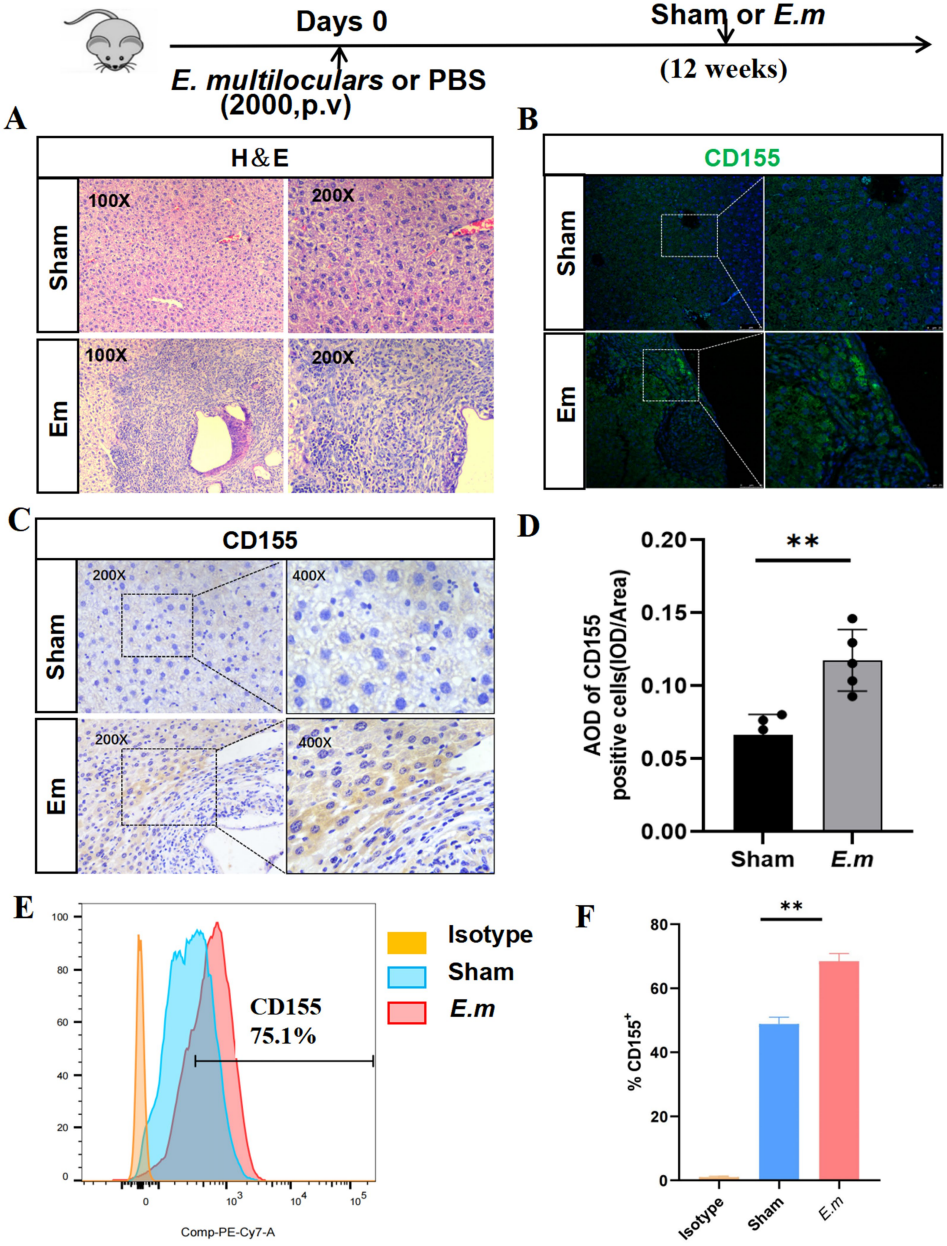
CD155 expression in the liver of the *E. multilocularis* infection mouse model. **(A)** H&E staining (*n* = 5). **(B)** Immunofluorescence staining of CD155 (*n* = 5). (**C)** IHC of CD155 (*n* = 5). **(D)** Statistical graphs of IHC. **(E)** Flow cytometry results of CD155 expression in primary hepatocytes (*n* = 5). **(F)** Statistical graphs of flow cytometry. *p < 0.05, **p < 0.01, ***p < 0.001.

To investigate the potential role of CD155 in AE progression, we first established the *E. multilocularis* infection mouse model. Compared to the Sham group, H&E staining results revealed significant infiltration of inflammatory cells and fibrotic tissues surrounding the lesions in the Em group (Figure 1A). Flow cytometry results showed that the percentage of CD155 + cells in the Em group was significantly elevated—approximately 1.5-fold that of the Sham group (Figures 1E,F). Furthermore, IHC and IF analyses demonstrated a significant elevation of CD155 expression in the Em group—nearly 2-fold that of the Sham group. These CD155-positive cells were predominantly hepatocytes adjacent to the lesions (Figures 1B–D). These results indicate that CD155 is upregulated in hepatocytes surrounding liver lesions in a mouse model of *E. multilocularis* infection.

### 3.2 CD155 expression correlates with T cell functional exhaustion in *Echinococcus multilocularis* infection

**Figure 2:**
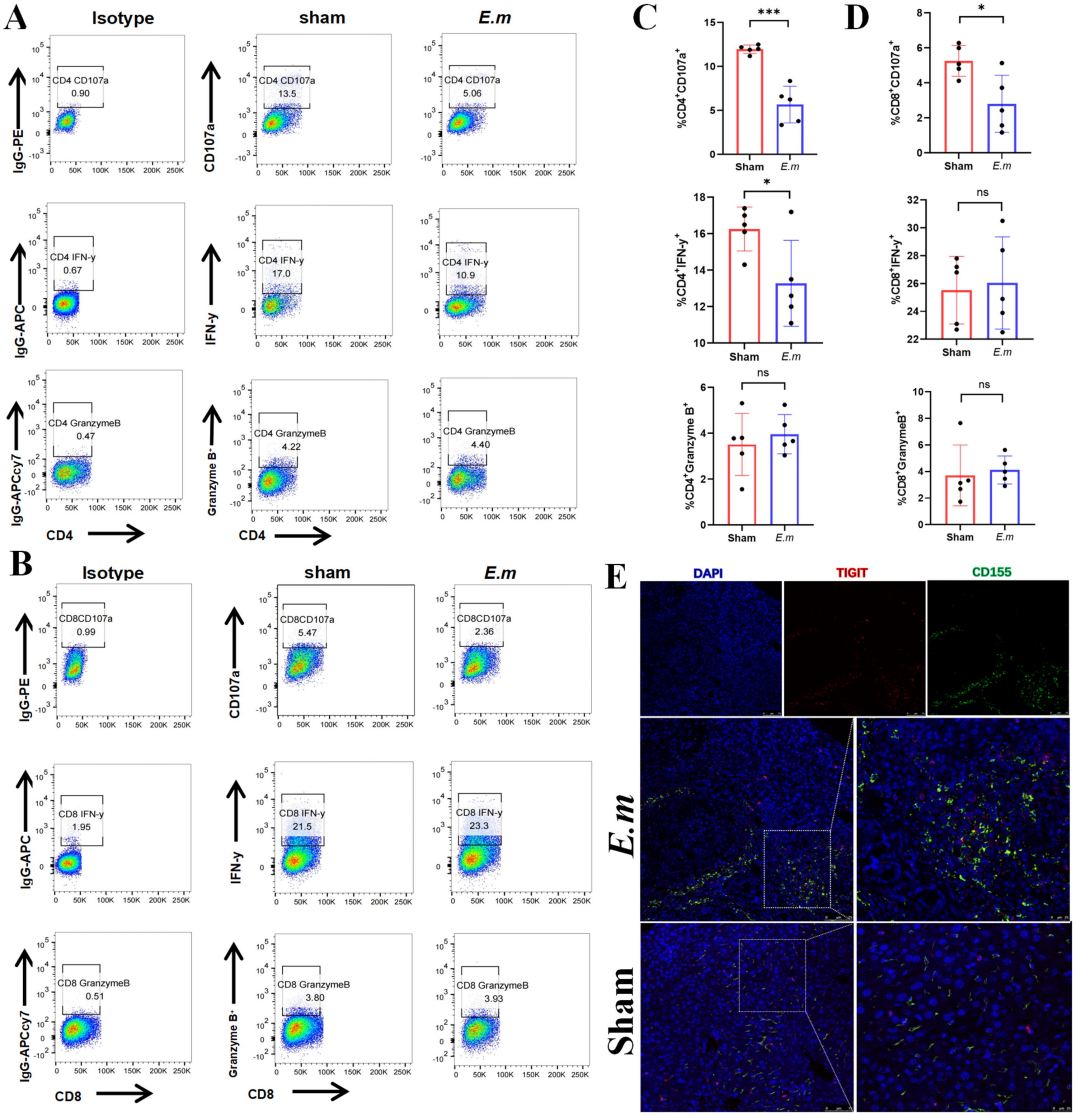
CD155 expression correlates with T cell functional exhaustion. **(A)** Flow cytometry results of CD107α, IFN-γ, and granzyme B in CD4^+^ T cells (*n* = 5). **(B)** Flow cytometry results of CD107α, IFN-γ, and granzyme B in CD8^+^ T cells (*n* = 5). **(C)** Statistical graphs of flow cytometry for CD4^+^ T cells (*n* = 5). **(D)** Statistical graphs of flow cytometry for CD8^+^ T cells (*n* = 5). **(E)** Multiple immunohistochemical staining of CD155 and TIGIT (*n* = 5). *p < 0.05, **p < 0.01, ***p < 0.001.

To elucidate the potential role of CD155 in T cell function within the *E. multilocularis* infection mouse model, we employed flow cytometry to assess CD4^+^ and CD8^+^ T cell function. The results indicated that the Em group exhibited a lower percentage of CD4^+^IFN-γ^+^, CD4^+^CD107α^+^, and CD8^+^CD107α^+^ cells than the sham group, but no significant difference was observed in the percentages of CD4^+^granzyme B^+^, CD8^+^IFN-γ^+^, and CD8^+^granzyme B^+^ cells between the two groups (Figures 2A–D). Besides, multiplex immunohistochemistry analysis revealed that both CD155 and TIGIT were upregulated in the Em group, with TIGIT^+^ cells being enriched around CD155^+^ cells (Figure 2E). These findings suggest that the elevated expression of CD155 may be associated with the functional exhaustion of infiltrating T cells in the *E. multilocularis* infection mouse model.

### 3.3 Metacestode vesicles promote CD155 upregulation in hepatocytes

**Figure 3:**
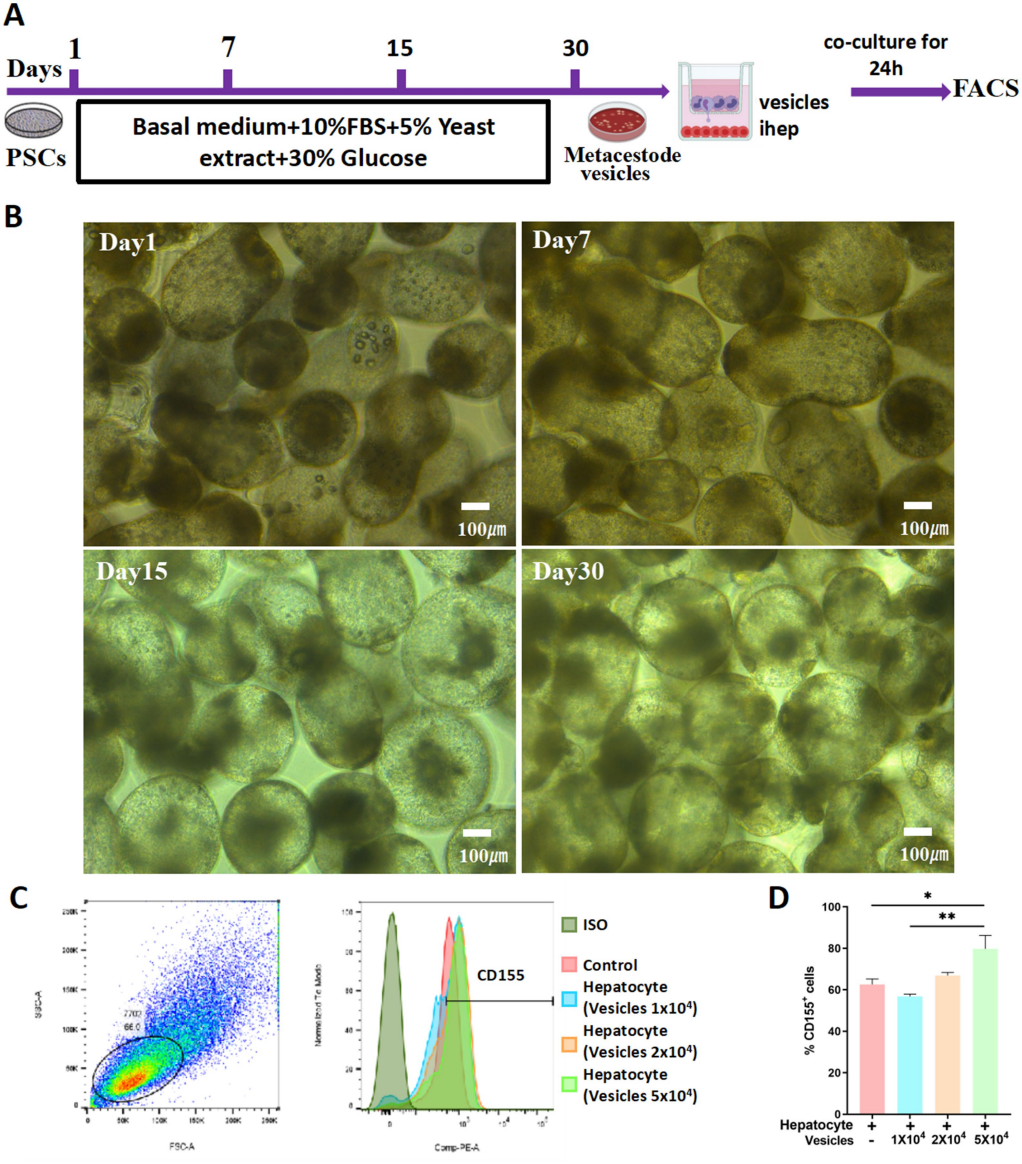
Metacestode vesicles promote CD155 upregulation in hepatocytes. **(A)** Schematic diagram. **(B)** Morphological changes from protoscoleces to hydatid cysts at different times. **(C,D)** Flow cytometry results of CD155 expression (*n* = 3). *p < 0.05, **p < 0.01, ***p < 0.001.

Metacestode vesicles play a crucial role in AE progression. To explore the potential correlation between metacestode vesicles and the differential expression of CD155 in hepatocytes, we first induced the formation of metacestode vesicles from protoscoleces *in vitro*. On Day 1, the protoscoleces exhibited vigorous motility, with some demonstrating eversion that revealed the rostellum and suckers. Under high-power microscopy, the small hook structure of the apical process was clearly visible, and the calcium particles within the somites were distinctly observable. By Day 7, the proglottids grew larger. On Day 15, the formation of metacestode vesicles was initiated, characterized by transparent, hypertrophied proglottids and rostellar hooks merging with the proglottids. By Day 30, the volume of the metacestode vesicles increased, while the hooks atrophied and detached, and the suckers regressed (Figures 3A,B). Subsequently, we co-cultured HL-7702 cells with metacestode vesicles and utilized flow cytometry to assess CD155 expression on HL-7702 cells. HL-7702 cells cultured alone (control group) showed low baseline expression of CD155, confirming their role as a negative control for comparison with co-culture conditions. The results revealed a dose-dependent increase in the percentage of CD155 + cells with escalating numbers of co-cultured metacestode vesicles (10,000, 20,000, and 50,000), reaching nearly 1.5-fold that of the control group at the highest concentration (Figures 3C,D). These findings suggest that excretory/secretory products from metacestode vesicles may play a role in regulating CD155 expression in hepatocytes.

### 3.4 Hepatocyte CD155 knockout restores T-cell function

**Figure 4:**
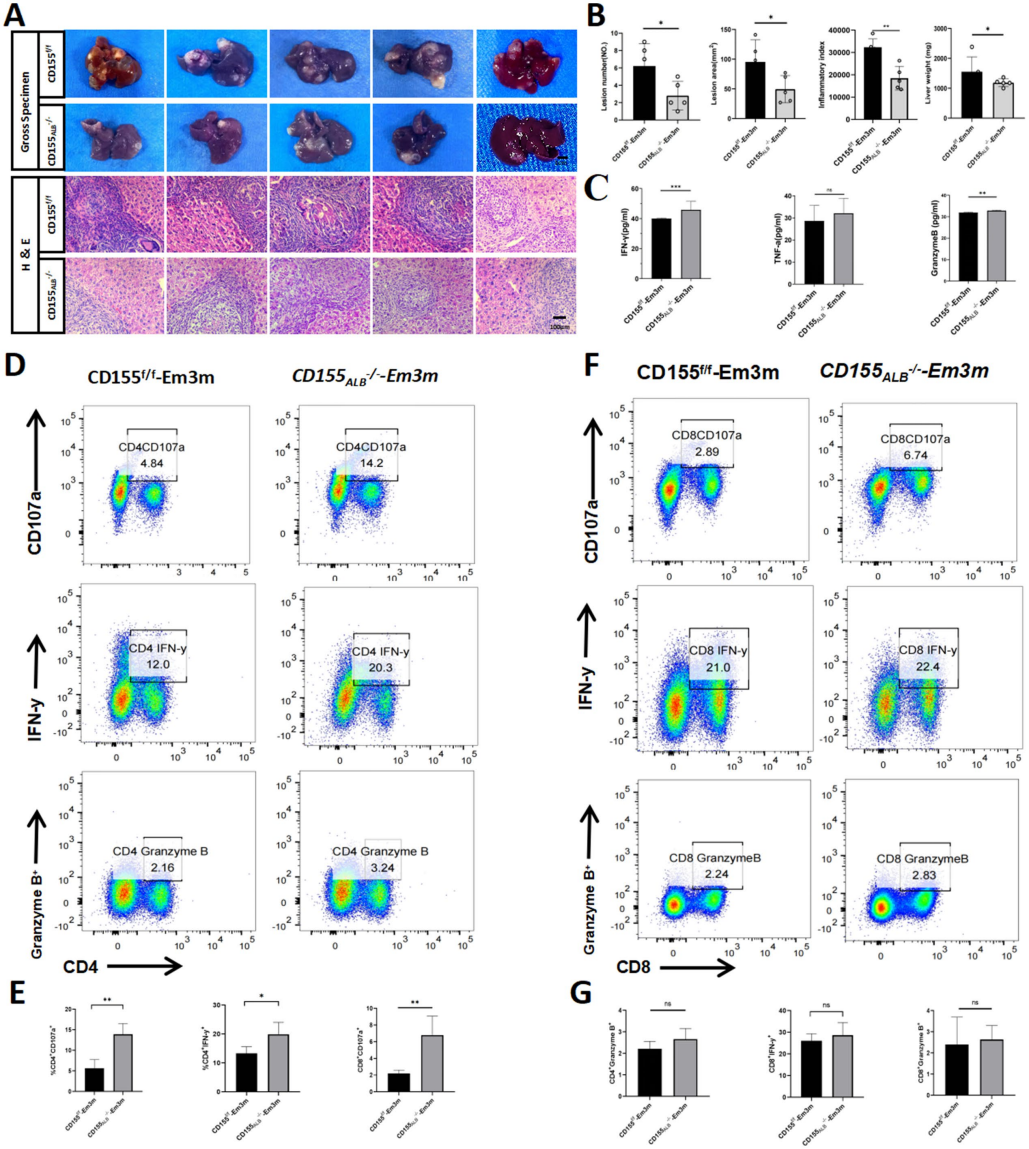
Hepatocyte CD155 knockout restores T cell function. **(A,B)** Statistical graphs of the inflammatory index, lesion area, and total liver weights (*n* = 5). **(C)** ELISA results of granzyme B, IFN-γ, and TNF-α (*n* = 5). **(D,E)** Flow cytometry plots of CD107α, IFN-γ, and granzyme B in CD4^+^ T cells (*n* = 5). **(F,G)** Flow cytometry plots of CD107α, IFN-γ, and granzyme B in CD8^+^ T cells (*n* = 5). *p < 0.05, **p < 0.01, ***p < 0.001.

To investigate the physiological significance of CD155 expression in T cell function, we established a CD155_Alb_^−/−^ mouse model infected with *E. multilocularis*. There were no significant differences in collagen deposition and hepatic stellate cell activation between the CD155^f/f^ group and the CD155_Alb_^−/−^ group (Supplementary Figures S2A,B). However, compared to the CD155^f/f^ group, the CD155_Alb_^−/−^ group exhibited a reduction in total liver weight, inflammatory index, lesion number, and lesion area (Figures 4A,B). Flow cytometry analyses indicated that the CD155_Alb_^−/−^ group exhibited a higher percentage of CD4^+^CD107a^+^, CD4^+^IFN-γ^+^, and CD8^+^CD107a^+^ cells in the liver than the CD155^f/f^ group, with no significant differences in the percentages of CD4^+^granzyme B^+^, CD8^+^IFN-γ^+^, and CD8^+^granzyme B^+^cells between the two groups (Figures 4D–G). Enzyme-linked immunosorbent assay (ELISA) results demonstrated that the CD155_Alb_^−/−^ group exhibited elevated serum levels of granzyme B and IFN-γ compared to the CD155^f/f^ group. Although TNF-α levels did not differ significantly between the two groups, a trend toward increased levels was noted in the CD155_Alb_^−/−^ group (Figure 4C). These findings suggest that hepatocyte-specific knockout of CD155 can restore the function of infiltrating T cells and reverse immune exhaustion in the *E. multilocularis* infection mouse model.

## 4 Discussion

The exhaustion of T cells enables *E. multilocularis* to evade host immune defenses, facilitating long-term parasitism. This study demonstrated that *E. multilocularis* induces T-cell exhaustion and accelerates disease progression by upregulating CD155 expression in hepatocytes. Targeting CD155 in hepatocytes may restore T-cell function and reverse T-cell exhaustion, suggesting that hepatocytes could emerge as a promising target for the immunotherapy of patients with AE.

AE is a life-threatening zoonotic parasitic disease characterized by chronic inflammation, granuloma formation, and extensive liver fibrosis (Wei et al., 2018; Wen et al., 2019; Reuter et al., 2004). This chronic infection often results in immune exhaustion, which plays a critical role in clinical challenges and poor prognosis. The fundamental mechanism of immune exhaustion stems primarily from the prolonged overactivation of immune checkpoints. These checkpoints, under normal circumstances, play crucial roles in regulating T cell function, preserving immune homeostasis, and preventing excessive immune responses (Sachdev et al., 2017; O’Donnell et al., 2018). However, excessive activation of these checkpoints can result in T-cell exhaustion. Key checkpoint molecules involved in this process include PD-1, CTLA-4, TIGIT, LAG-3, and TIM-3 (MacDonald et al., 2016). Our previous studies revealed significantly elevated expression of the immune inhibitory receptor TIGIT on hepatic T cells in patients with AE, leading to T-cell exhaustion and promoting the survival of the *E. multilocularis* metacestode (Zhang et al., 2020). CD155, the primary high-affinity ligand for TIGIT, inhibits T-cell function upon interaction (Zhang et al., 2021). Based on these findings, we directed increased attention toward the upstream ligand of TIGIT, CD155, aiming to dissect its specific role—particularly that of hepatocyte-derived CD155—in shaping the immunosuppressive microenvironment. In this study, we observed significant upregulation of CD155 expression on hepatocytes surrounding lesions in the *E. multilocularis* infection mouse model, highlighting its crucial role in disease progression. Furthermore, we observed significant impairment of CD4^+^ and CD8^+^ T cell function, with TIGIT^+^ cells being notably enriched near CD155^+^ cells in the livers of infected mice. These findings suggest that *E. multilocularis* infection induces hepatocyte CD155 expression, potentially leading to CD4^+^ and CD8^+^ T cell exhaustion through intercellular interactions.

The *E. multilocularis* infection results in the formation of tumor-like masses in the host liver, characterized by numerous vesicles of varying sizes that play a crucial role in infection pathogenesis (McManus et al., 2003). The lack of a fibrous tissue capsule allows direct contact between metacestode vesicles and host tissue, promoting aggressive invasion and growth, as well as significant pathological effects due to the exposure of host tissue to excretory/secretory products (Herz and Brehm, 2021). These excretory/secretory products from metacestode vesicles can induce apoptosis in dendritic cells, increase the population of CD4^+^CD25^+^Foxp3^+^ regulatory T cells, and inhibit the function of mononuclear cells (MacDonald et al., 2012; Hübner et al., 2006). In this study, we observed a significant upregulation of CD155 expression on the surface of hepatocytes co-cultured with metacestode vesicles. This finding suggests that the excretory/secretory products from metacestode vesicles may induce CD155 expression in hepatocytes, thereby contributing to an immunosuppressive microenvironment that facilitates the immune evasion of parasites during infection.

CD155 is emerging as a potential target for immunotherapy due to its critical role in disease progression, including various tumors and infections (Paolini and Molfetta, 2023). Studies have demonstrated that CD155 is not only highly expressed in numerous tumors but also closely associated with poor prognostic outcomes (Martinez-Ortega et al., 2023; Ma et al., 2023). Within the tumor microenvironment, CD155 modulates the function of T cells and natural killer cells by interacting with inhibitory receptors, thereby playing a critical role in the immune evasion of tumor cells (Cho et al., 2023). Therapeutic strategies targeting CD155 have demonstrated potential in reversing T-cell exhaustion and enhancing antitumor responses. In this study, we knocked out CD155 in hepatocytes, blocking its interaction with receptors, which led to the reversal of the exhaustion state of CD4^+^ and CD8^+^ T cells, as well as the immunosuppressive state of the liver in an *E. multilocularis* infection mouse model. This intervention significantly reduced both the number and size of lesions and alleviated the progression of *E. multilocularis* infection. Consequently, CD155 represents a critical therapeutic target with the potential to reverse immune exhaustion and control the progression of *E. multilocularis* infection.

Although our findings demonstrate that hepatocyte-specific CD155 knockout reverses immune exhaustion and mitigates lesion progression in a murine model of *E. multilocularis* infection, several limitations should be acknowledged. First, although the mouse model used in this study is well-established, it cannot fully recapitulate the complexity of human AE pathology and immune responses. Species-specific differences, particularly in the regulation of immune checkpoint pathways such as the TIGIT–CD155 axis, may affect the translational relevance of our findings. Second, although we performed additional histological analyses, including H&E, Masson staining, and α-SMA immunohistochemistry, to demonstrate that hepatocyte-specific CD155 deletion does not influence collagen deposition or hepatic stellate cell activation, the broader physiological effects and long-term safety of this genetic intervention remain to be further investigated. Third, while our study revealed a clear association between CD155 expression and T-cell exhaustion, the underlying molecular signaling pathways responsible for CD155 upregulation and its immunoregulatory effects were not explored in detail. Future studies employing transcriptomic or proteomic profiling may help to elucidate these mechanisms and provide additional therapeutic targets.

## 5 Conclusion

**Figure 5:**
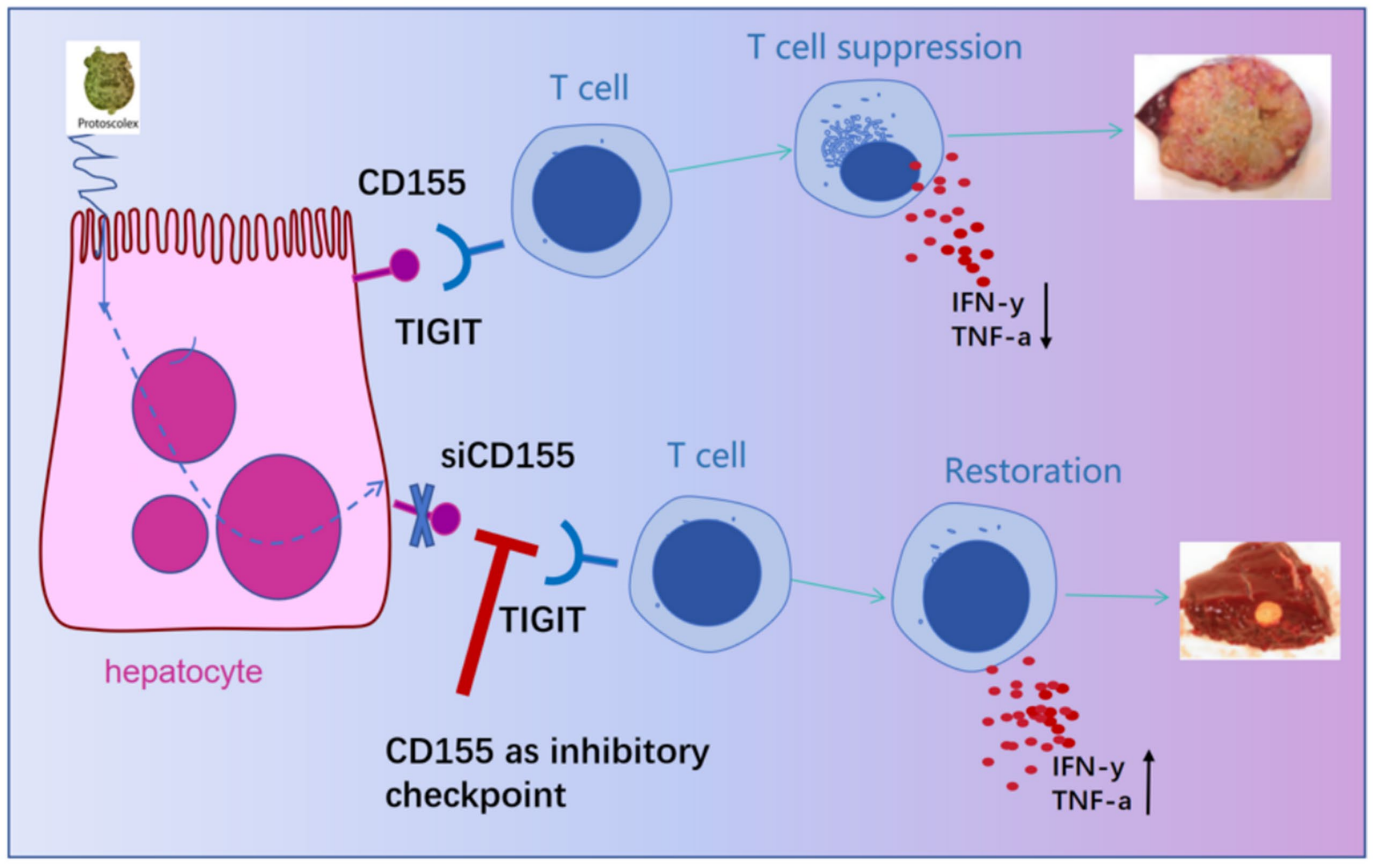
Schematic diagram of the study on the functional inhibition and targeted intervention of T cells by the interaction between liver cell CD155 and TIGIT.

In summary, this study reveals that excretory/secretory products from metacestode vesicles induce CD155 expression in hepatocytes, fostering an immunosuppressive microenvironment that drives immune exhaustion during *E. multilocularis* infection (Figure 5). Hepatocyte-specific CD155 knockout reversed T-cell exhaustion, restored CD4^+^ and CD8^+^ T-cell functionality, and significantly reduced lesion size and number in the infection mouse model. These findings identify a novel immune evasion mechanism and establish CD155 as a promising therapeutic target for AE, warranting further exploration of its clinical potential and its combination with existing immunotherapies.

## Data Availability

The raw data supporting the conclusions of this article will be made available by the authors, without undue reservation.

## Supplementary material

Supplementary FIGURE S1 Genotype identification of the CD155^f/f^-Alb-CreERT2 mice. **(A)** Agarose Gel Electrophoresis: The length of the mutant gene segment is 366 bp; the length of the wild-type gene segment is 250 bp; the length of the Cre gene fragment is 350 bp. **(B)** IHC of CD155 (*n* = 3). **(C)** Flow cytometry of CD155 (*n* = 3). **p* < 0.05, ***p* < 0.01, ****p* < 0.001.

Supplementary FIGURE S2 Evaluation of the side effects of hepatocyte-specific CD155 deletion. **(A,B)** Masson staining, IHC of a-SMA and statistical graphs (*n* = 5). **p* < 0.05, ***p* < 0.01, ****p* < 0.001.
