# Supplementary figures and images for: CD155 as a therapeutic target in alveolar echinococcosis: insights from an Echinococcus multilocularis infection mouse model
Source: Front Microbiol. 2025 Jul 1;16:1624387. doi: 10.3389/fmicb.2025.1624387 (PMC12259647; doi:10.3389/fmicb.2025.1624387)

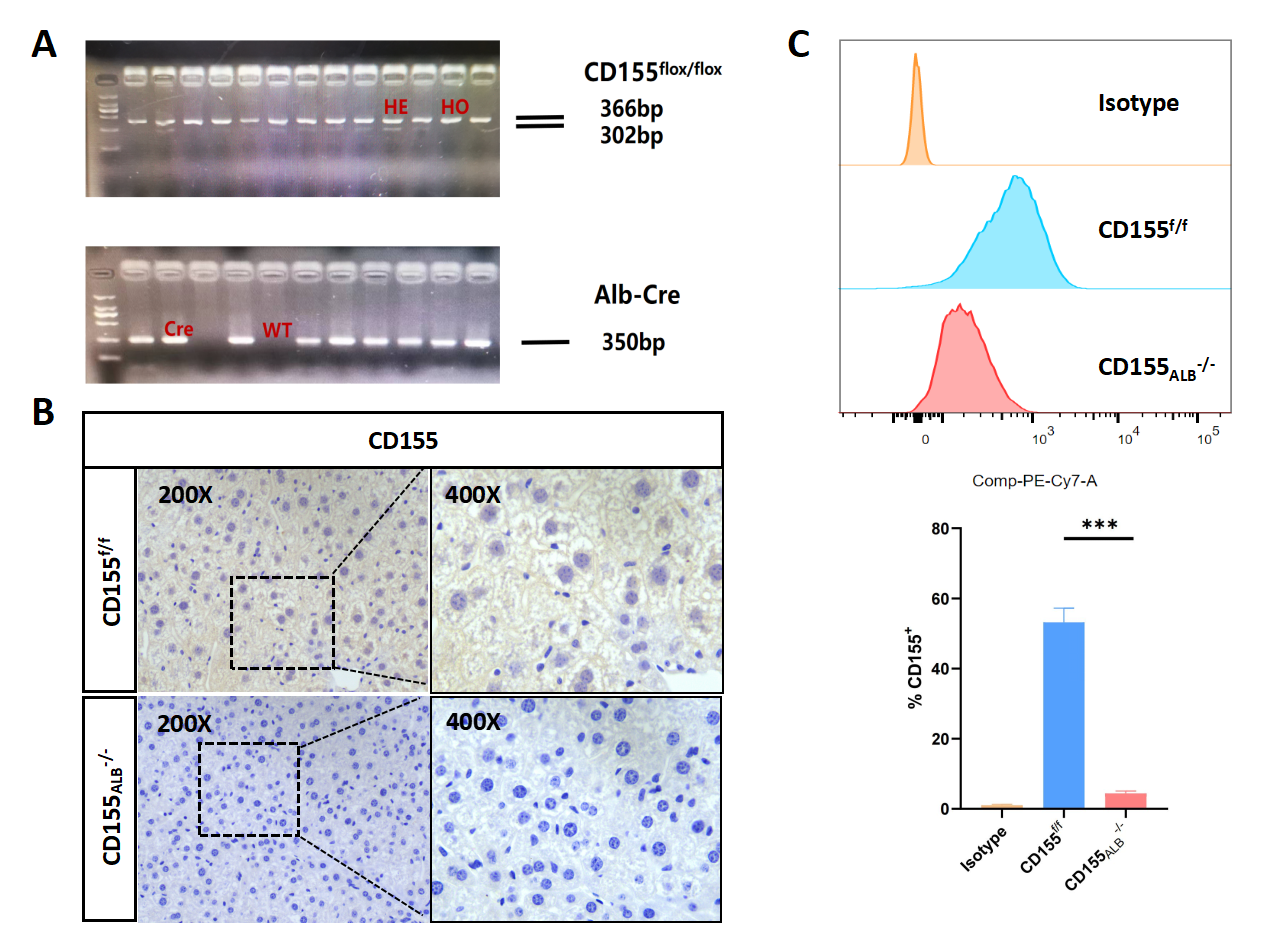

Supplement: Supplementary FIGURE S1 — Genotype identification of the CD155f/f-Alb-CreERT2 mice. (A) Agarose Gel Electrophoresis: The length of the mutant gene segment is 366 bp; the length of the wild-type gene segment is 250 bp; the length of the Cre gene fragment is 350 bp. (B) IHC of CD155 (n = 3). (C) Flow cytometry of CD155 (n = 3). *p < 0.05, **p < 0.01, ***p < 0.001. [file Image_1.tif]

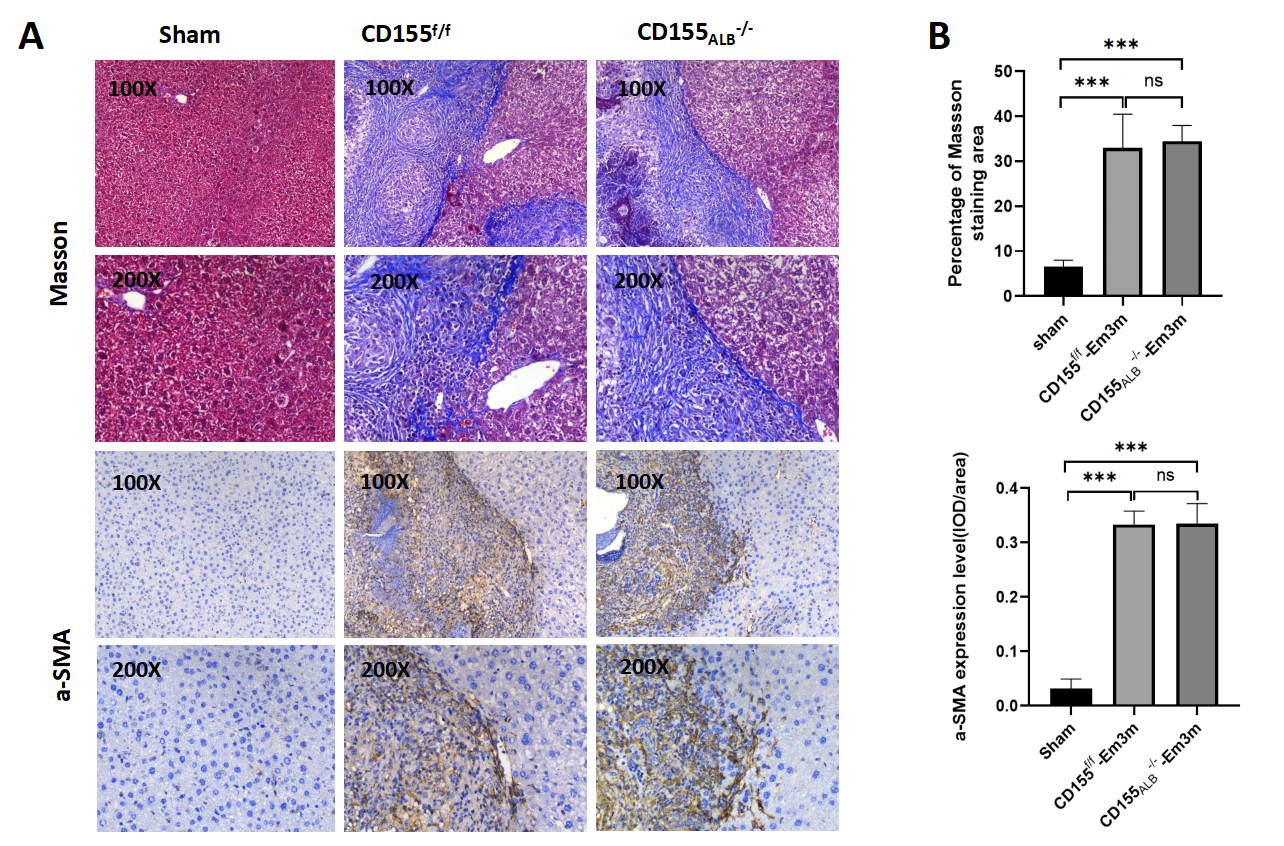

Supplement: Supplementary FIGURE S2 — Evaluation of the side effects of hepatocyte-specific CD155 deletion. (A,B) Masson staining, IHC of a-SMA and statistical graphs (n = 5). *p < 0.05, **p < 0.01, ***p < 0.001. [file Image_2.tif]
